# 9-Hydroxyaristoquinolone: A New Indole Alkaloid Isolated from *Aristotelia chilensis* with Inhibitory Activity of NF-κB in HMC-3 Microglia Cells

**DOI:** 10.3390/ijms26062419

**Published:** 2025-03-07

**Authors:** Rebeca Pérez, Viviana Burgos, Jaime R. Cabrera-Pardo, Leandro Ortiz, Antoni Camins, Miren Ettcheto, Bernd Schmidt, Vaderament-A. Nchiozem-Ngnitedem, Cristian Paz

**Affiliations:** 1 Laboratory of Natural Products & Drug Discovery, Center CEBIM, Department of Basic Sciences, Faculty of Medicine, Universidad de La Frontera, Temuco 4780000, Chile; perezcolladorebeca@gmail.com; 2 Escuela de Tecnología Médica, Facultad de Salud, Universidad Santo Tomás, Temuco 4780000, Chile; vburgos7@santotomas.cl; 3 Laboratorio de Química Aplicada y Sustentable (LabQAS), Departamento de Química, Universidad del Bío-Bío, Avenida Collao 1202, Concepcion 4051381, Chile; jacabrera@ubiobio.cl; 4 College of Dental Medicine, Roseman University of Health Sciences, 10894 S. River Front Parkway, South Jordan, UT 84095, USA; 5 Instituto de Ciencias Químicas, Facultad de Ciencias, Universidad Austral de Chile, Valdivia 5110566, Chile; leandro.ortiz@uach.cl; 6 Departament de Farmacologia, Toxicologia i Química Terapèutica, Facultat de Farmàcia i Ciències de l’Alimentació, Universitat de Barcelona (UB), Av. de Joan XXIII, 27-31, 08028 Barcelona, Spain; camins@ub.edu (A.C.); mirenettcheto@ub.edu (M.E.); 7 Institut de Neurociències, Universitat de Barcelona (UB), Passeig de la Vall d’Hebron, 171, 08035 Barcelona, Spain; 8 Centro de Investigación Biomédica en Red Enfermedades Neurodegenerativas (CIBERNED), Instituto de Carlos III, Av. Monforte de Lemos, 3-5, 28029 Madrid, Spain; 9 Institut d’Investigació Sanitària Pere Virgili (IISPV), Hospital Universitari Sant Joan de Reus, Av. Josep Laporte, 2, 43204 Reus, Spain; 10 Institut für Chemie, Universität Potsdam, Karl-Liebknecht-Str. 24-25, D-14476 Potsdam, Germany; bernd.schmidt@uni-potsdam.de (B.S.); n.vaderamentalexe@gmail.com (V.-A.N.-N.)

**Keywords:** *Aristotelia chilensis*, quinoline alkaloid, NF-κB, microglial cell HMC-3

## Abstract

Neurodegenerative diseases are characterized by a progressive process of degeneration and neuronal death in the nervous system, with neuroinflammation being one of the main factors contributing to the progression of these diseases. *Aristotelia chilensis* (Maqui) is a native tree of Chile used in the Mapuche folk medicine for wounds and digestive treatment. It produces edible black berries with the highest antioxidant capacity among berries, and the Mapuche people used it for producing an alcoholic beverage. The leaves of Maqui contain indole alkaloids with different pharmacological properties that suggest neuroprotective effects. Here, the isolation and chemical characterization of a new alkaloid, named 9-hydroxyaristoquinolone, and the evaluation of its anti-inflammatory activity in the microglial cell line HMC-3, treated with LPS, are reported. 9-Hydroxyaristoquinolone protects microglia from LPS-induced morphological changes at concentrations as low as 1 µM, with a reduction in IKBα-P levels and inhibition of the NF-κB pathway, which was assessed by THP-1 NF-κB dual cell reporter and Western blot in HMC-3 cells. In silico studies suggest that 9-hydroxyaristoquinolone does not induce hepatotoxicity or genotoxicity and exhibits BBB permeability.

## 1. Introduction

Medical advances and public health initiatives have succeeded in reducing mortality, increasing global life expectancy to 73.4 years in 2023, with projections that it will reach 77.2 years by 2050 [1,2]. However, given that aging is the main risk factor for neurodegenerative diseases, the increasing proportion of older adults in the population implies a greater risk of developing these pathologies as a direct consequence of longer survival [3]. Alzheimer’s disease (AD), the most prevalent form of dementia, currently affects about 50 million people worldwide, and this number is expected to triple by 2050 [4].

Despite decades of clinical trials based on the amyloid cascade theory, the results have been unsuccessful [5]. Currently, research highlights the crucial role of neuroinflammation in the pathogenesis of neurodegenerative diseases [6]. In AD, there is a tipping point along its pathophysiological evolution where glial cells maintain an overexpressed inflammatory response that synergizes with amyloid-β (Aβ) and tau accumulation and drives synaptotoxicity and neurodegeneration in a self-reinforcing manner [7]. Amyloid compaction exposes microglia to Aβ and contributes to microglial activation through Toll-like receptors (TLRs) and NOD-like receptor signaling, leading to the production of proinflammatory cytokines [8,9]. Proinflammatory cytokines released by activated microglia reduce the expression of Aβ-binding receptors and Aβ-degrading enzymes, leading to further accumulation of Aβ. Although microglia can phagocytose tau protein [10], they cannot degrade it and may even contribute to tau seeding [11].

In 2008, Patel et al. demonstrated that the expression of some receptors such as tumor necrosis factor receptor 2 (TNFR2) is upregulated by changes that are related to increasing age [12]. Binding of tumor necrosis factor (TNF-α) to the TNFR2 receptor (75 kDa TNF 2) promotes neuroprotective and regenerative pathways through interaction with a number of TNF-α receptor-associated factors and cellular inhibitory proteins of apoptosis, leading to excessive activation of Mitogen-Activated Protein Kinases (MAP kinases) and the Akt and nuclear factor kappa-light-chain-enhancer of activated B cells (NF-ΚB) pathways [11,13]. Moreover, Aβ peptide was shown to bind to TLRs and activate IκB kinase (IKK), resulting in phosphorylation of inhibitory κB (IκB) and release of NF-κB, the **master** transcription factor that regulates the production of proinflammatory cytokine precursors such as interleukin (IL)-1β, IL-6, IL-18, TNF-α and cyclooxygenase-2 (COX2), of chemokines such as the CC motif chemokine ligand 1 (CCL1), CCL5 and CXC motif ligand 1 (CXCL1), of small molecule messengers (prostaglandins, nitric oxide, reactive oxygen species) and NOD-, LRR- and Pyrin domain-containing protein 3 (NLRP3) inflammasome assembly. Caspase-1, contained in the NLRP3 inflammasome, cleaves precursors of proinflammatory cytokines, resulting in IL-1β and IL-18, which can damage neurons [14,15,16]. Therefore, modulation of the NF-κB pathway is considered a promising strategy for the development of neuroprotective therapies [17,18].

*Aristotelia chilensis,* commonly known as Maqui, is a plant native to Chile widely used in traditional medicine for its antioxidant, anti-inflammatory and hepatoprotective properties. In recent years, it has aroused great interest in scientific research due to the diversity and potential of its bioactive compounds. Maqui is known worldwide for its extraordinary antioxidant properties found mainly in the fruit. On the other hand, its leaves have been found to have important analgesic, anti-inflammatory, antioxidant, antiviral and α-glucosidase inhibitory activities [19,20,21]. Among the components of its leaves are non-iridoid monoterpene indole alkaloids, such as aristoteline, aristotelone, aristone, aristoquinoline (**1**), hobartine (**2**), 8-oxohobartine and 8-oxo-9-dehydromakomakine. Some of these metabolites display antioxidant, vasodilator and acetylcholinesterase antagonist activity, and the ability to modulate inflammatory responses, suggesting that they may have protective effects in the context of neurodegenerative diseases [22,23,24,25].

Herein, the isolation of a new indole alkaloid, called 9-hydroxyaristoquinolone (**3**), from the leaves of *Aristotelia chilensis* and its structure elucidation by high-field NMR and HRMS methods was investigated. The anti-inflammatory activity profile and inhibitory effect on the NF-κB pathway of this new secondary metabolite were studied, with the result that this alkaloid reduces the phosphorylation of IκBα and its translocation to the nucleus, reducing the NF-κB activation by LPS.

## 2. Results

### 2.1. Chemical Characterization of 9-Hydroxyaristoquinolone (**3**)

**Figure 1:**
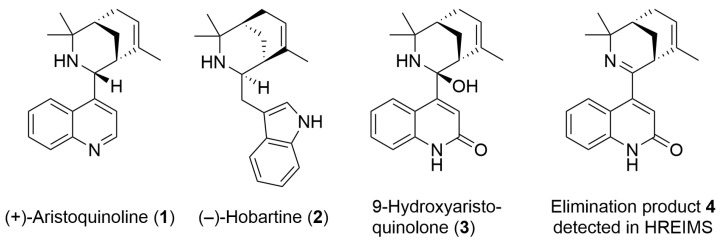
Structures of known *Aristotelia* alkaloids aristoquinoline (**1**), hobartine (**2**), the new compound 9-hydroxyaristoquinolone (**3**) and the elimination product **4** detected by HREIMS.

9-Hydroxyaristoquinolone (**3**) was isolated as a brown oil. The FT-ATR-IR indicates the presence of N-H and O-H groups and a carboxamide group (*v* 1661 cm^−1^, s). The HREIMS ([M^+^] *m*/*z* 306.1723) points at a molecular formula of C_20_H_22_N_2_O (calc 306.1732), which indicates eleven double bond equivalents. The ^13^C-NMR spectrum shows 20 signals: one at *δ*_C_ 174.0 ppm (in agreement with a C=O (NHR) moiety), four signals for quaternary C-atoms in the aromatic/olefinic region, six signals for sp^2^-CH- groups in the aromatic/olefinic region, one signal for a quaternary carbon atom at *δ*_C_ 91.3 (indicative for an sp^3^-C-atom bound to one N- and one O-atom), one signal for a quaternary carbon atom at *δ*_C_ 58.9 (indicative for an amine), two signals for C-H-groups, two signals for CH_2_-groups, and three signals for CH_3_-groups in the aliphatic region. This information points at six degrees of unsaturation and at four ring closures, which adds up to ten double bond equivalents. This apparently contradicts the information gathered from the HREIMS, which indicates eleven double bond equivalents. It was therefore assumed that, rather than the actual [M^+^] peak, the [M-H_2_O]^+^ peak was observed in the HREIMS (**4** in Figure 1), and that the actual molecular formula of 9-hydroxyaristoquinolone is thus C_20_H_24_N_2_O_2_. The total number of carbon atoms, the presence of two nitrogen atoms and the ^1^H-NMR spectroscopical data suggest that 9-hydroxyaristoquinolone is structurally related to the *Aristotelia* alkaloids aristoquinoline (**1**) and hobartine (**2**) (Figure 1) [22,26,27,28,29]. HMBC correlations between H-5 (*δ*_H_ 7.44 ppm) and C-4 (*δ*_C_ 162.0 ppm), H-3 (*δ*_H_ 5.90 ppm) and C-2 (174 ppm), C-4 (162 ppm) and C-9 (91.3 ppm), and, in particular, the absence of an aliphatic CH_2_-group for C-4 indicate that 9-hydroxyaristoquinolone is a quinolone-derivative, rather than an indole as in hobartine (**2**). Another striking difference between aristoquinoline (**1**) and hobartine (**2**) is the absence of a signal for a proton H-9 at ca. *δ*_H_ 3.5 ppm and a methine carbon C-9 at ca. *δ*_C_ 55 ppm [27]. Instead, C-9 is a quaternary carbon at *δ*_C_ 91.3 ppm, which indicates that C-9 is sp^3^-hybridized and connected to N-10 and a hydroxy group. A NOE interaction between H-17 (the methyl group bound to C-16) and H-3 and H-5 points at an axial orientation of the C-9-OH group, and hence an equatorial orientation of the quinolone substituent attached to C-9. This configuration at C-9 is the same as in other *Aristotelia* alkaloids [26]. The fact that under EI-MS conditions, no [M^+^]-peak, but a signal at *m*/*z* 306.1723 ([M-H_2_O]^+^) was observed suggests a facile elimination of water with formation of an imine **4** (Figure 1). An analogous imine is an intermediate in a total synthesis of aristoquinoline (**1**) published by Riley and co-workers [20]. It should be noted that oxygenation at C2 and at C9 is, to the best of our knowledge, unprecedented for *Aristotelia* alkaloids. Table 1 summarizes NMR data and relevant HMBC and NOE correlations for 9-hydroxyaristoquinolone (**3**), Figures S1–S6.

### 2.2. Analytical Data for 9-Hydroxyaristoquinolone

9-Hydroxyaristoquinolone (**3**). Brown oil; ${\left[\alpha \right]}_{D}^{26}$ + 7.3 (*c* 0.175, CH_2_Cl_2_); IR (ATR) *ν̃* 3317, 2917, 2850, 1660, 1462 cm^−1^; ^1^H and ^13^C NMR see Table 1; HREIMS [M-H_2_O]^+^ *m*/*z* 306.1723 (calcd for C_20_H_22_N_2_O, 306.1732).

### 2.3. Evaluation of the Pharmacokinetic and Toxicity Profiles of 9-Hydroxyaristoquinolone by In Silico

The study of the pharmacokinetic profiles of 9-hydroxyaristoquinolone using the SwissADME online server and the ProTox-III platform “https://tox.charite.de/protox3/ (accessed on 14 October 2024) highlights favorable characteristics as a neuroprotective agent. With a molecular weight of 324.424 Da and TPSA (polar surface area accessible) values of 65.12 Å^2^, the molecule meets the parameters for adequate biological permeability, especially considering its high gastrointestinal (GI) absorption and permeability across the blood–brain barrier (BBB). It does not show significant inhibition of the main cytochrome P450 enzymes (CYP1A2, CYP2C9, CYP2C19 and CYP3A4), which suggests a low potential for pharmacological interactions, as well as a good balance between hydrophobicity and hydrophilicity (Table 2).

### 2.4. Kinetic Assays of Morphological Changes by Live Cell AnalysisIncucyte^®^: 9-Hydroxyaristoquinolone Inhibits LPS-Induced Microglial Reactivation

The morphological study of the HMC-3 microglia cell line was performed with Incucyte, providing accurate and meaningful results through kinetic assays using super-resolution real-time live cell microscopy. Among the parameters that have been highlighted in the morphological study of LPS-activated microglia are Phase Area Confluence (indicating the amount of area occupied by microglia cells in response to LPS treatment) and Phase Object Count per Well (representing the total count of objects identified as microglia cells in a well).

**Figure 2:**
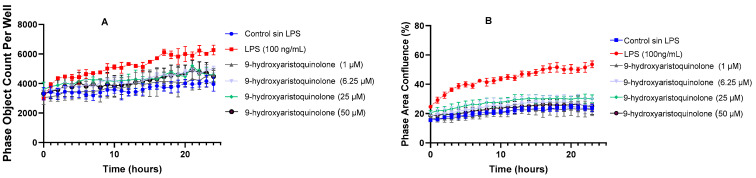
The IncuCyte^®^ live-cell analysis system was used to study the effect of 9-hydroxyaristoquinolone treatment on LPS-induced activation in the HMC-3 cell line. (**A**) Real-time graphs of Phase Object Count per Well measurements (24 h) in cells exposed to 1–50 μM of 9-hydroxyaristoquinolone. (**B**) Real-time graphs of Phase Area Confluence measurements (24 h) in cells exposed to 1–50 μM of 9-hydroxyaristoquinolone. Data are presented as mean ± SD (*n* = 3).

Measurement of the Phase Area Confluence parameter (%) over 24 h revealed the impact of incubation of HMC-3 cells with LPS, demonstrating a significant increase in confluence. This finding suggests an increased cell density or a change in cell expansion in response to microglia activation. On the other hand, co-treatment with 9-hydroxyaristoquinolone at concentrations starting at 1 µM together with LPS exhibited a neuroprotective effect, as they prevented HMC-3 cells from experiencing an increase in cell density, maintaining similar percentages to the control group, as shown in Figure 2B.

Additionally, it was observed that the reduction in cell density induced by 9-hydroxyaristoquinolone did not correlate with a decrease in the number of cells per well, as assessed over time by measuring the Phase Object Count per Well parameter. Our results indicate that there was no evidence of a reduction in cell count over time compared to the control group. We also note that the control group treated with LPS generated a higher proliferation in the HMC-3 cell population, as shown in Figure 2A.

### 2.5. Detection of NF-kB-SEAP Levels:9-Hydroxyaristoquinolone Reduces NF-KB Pathway Activity in THP-1 Reporter Cell

**Figure 3:**
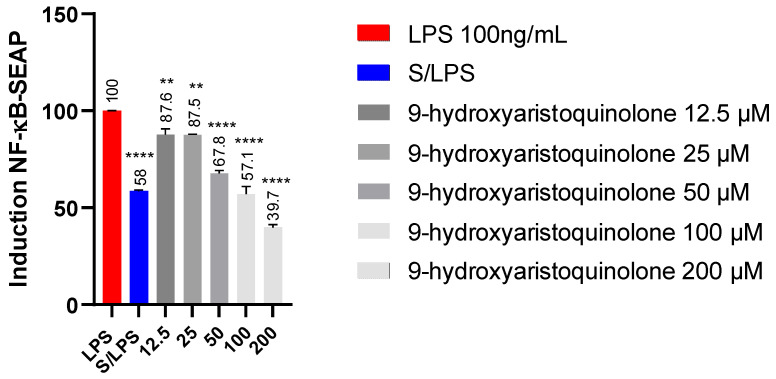
Effects of 9-hydroxyaristoquinolone on the induction of NF-κB-SEAP. THP1-DualTM cell was treated with compound 12.5–200 μM and LPS (100 ng/mL) for 18 h and assessed by THP-1-DualTM cell assay compared to positive control (LPS 100 ng/mL). The data were expressed as mean ± standard deviation. ANOVA, Dunnett’s Multiple Comparison Test (** *p* =< 0.01 and **** = *p* < 0.0001 vs. LPS (+) group).

Concentrations of 12.5, 25, 50, 50, 100 and 200 µM of 9-hydroxyaristoquinolone were used to evaluate its effect on modulatory activation of the NF-κB signaling pathway. In the experiment, cell culture supernatants were collected and subjected to spectrometric evaluation of the level of secreted embryonic alkaline phosphatase (SEAP). It was found that THP-1XBlue monocytes stimulated with LPS (100 ng/mL) produced a high level of SEAP, indicating activation of the nuclear transcription factor NF-κB. This SEAP activation was decreased by 9-hydroxyaristoquinolone. Concentrations of 12.5 and 25 µM also induced less high levels of SEAP than the control, but not enough to be significant. However, concentrations above 50 µM significantly decreased SEAP stimulation by LPS. 9-hydroxyaristoquinolone at 200 µM had the strongest effect, reducing SEAP activity by 51.3%, Figure 3.

### 2.6. Nuclear Factor NF-κB Modulation Assay: 9-Hydroxyaristoquinolone Inhibits LPS-Induced Phosphorylation of IκB-α

**Figure 4:**
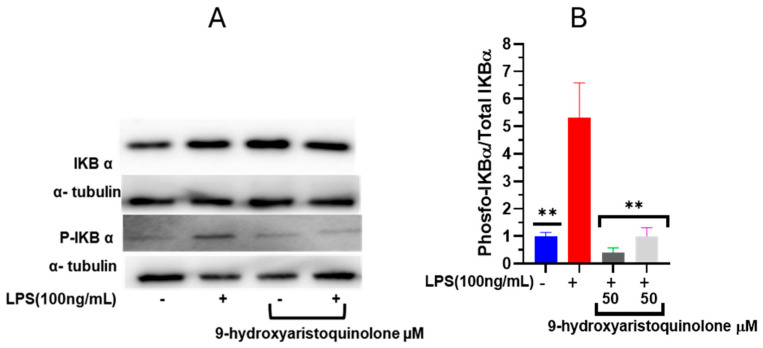
Effect of 9-hydroxyaristoquinolone on the protein expression of IkB-α/p-IkB-α on stimulated HMC-3 cells with LPS. HMC-3 cells were treated with 9-hydroxyaristoquinolone at 50 µM and LPS (100 ng/mL) for 2 h. (**A**) Western blot analysis normalized to the positive control group (LPS 100 ng/mL). (**B**) Normalized Protein expression of IkB-α/p-IkB-α.Data were expressed as mean ± standard deviation. ANOVA, Dunnett’s Multiple Comparison Test (** = *p* < 0.01 vs. LPS (+) group).

Nuclear factor NF-κB is an important transcriptional regulator that can induce the transcription of proinflammatory cytokines. Accordingly, the influence of 9-hydroxyaristoquinolone on the activation of this pathway was investigated. Free NF-κB dimer-activated subunits (p50/p65) can translocate from the cytosol to the nucleus after dissociation of IκB-α from NF-κB. The levels and phosphorylation of cytoplasmic IκBα proteins were detected by Western blot analysis. Figure 4 indicates that LPS induction significantly increased the expression of phosphorylated IκBα (IκBα-P). Pretreatment with 9-hydroxyaristoquinolone prevented the expression of IκBα-P. After pretreatment with 50 µM 9-hydroxyaristoquinolone, the ratio of P-IκBα/IκBα in HMC-3 cells decreased by 90%. Therefore, activation of the NF-κB inflammatory pathway can be suppressed by 9-hydroxyaristoquinolone in LPS-induced HMC-3 cells.

## 3. Discussion

The native Chilean plant *Aristotelia chilensis*, popularly known as Maqui, has been widely studied for its beneficial health properties, mainly attributed to its fruits. Its purple-black berries are known worldwide for their extraordinary antioxidant and bioactive properties [20,30,31]. Compared to other berries, Maqui contains significant amounts of flavonoids, especially anthocyanins, which possess neuroprotective activity due to their antioxidant and anti-inflammatory properties [32].

Maqui leaves contain a large amount of bioactive ingredients. Rubilar et al. (2011) and Muñoz et al. (2011) demonstrated that the leaves of this Chilean plant have an even higher total polyphenol content and antioxidant capacity than its berries [19,20]. Maqui leaves contain indole and quinolinic alkaloids (aristoteline, serratoline, aristone, horbatine, horbatinol, protopine, aristoquinoline, 3-formylindole), a group of compounds that, although less studied than its anthocyanins, have shown promising therapeutic potential. For example, the aqueous, ethanolic and methanolic extracts of its leaves have anti-inflammatory and analgesic properties [19] and antioxidant potential, with applications in cellular protection against oxidative stress and inflammation [19]. They also inhibit enzymes such as cholinesterase and tyrosinase, supporting the possible neuroprotective effects attributed to Maqui [33]. The indole alkaloids isolated from Maqui modulate nociceptive ion channels, including Nav 1.7 and Nav 1.8 [24]. This bioactivity profile justifies the new exploration of alkaloids from this plant, whose specific effects have not yet been widely evaluated. The new alkaloid 9-hydroxyaristoquinolone (**3**) is an oily compound that has been isolated in a yield of 0.00016%. It is structurally related to the alkaloid aristoquinoline (**1**) but is oxidized at C-2 of the quinoline moiety (carboxamide group, evidenced by *δ*_C-2_ = 174 ppm, *v* 1661 cm^−1^). Position 3 is an olefin (*δ*_H_ = 5.90 (s), *δ*_C-3_ = 117.9 ppm) and shows long-range C-H-couplings (evidenced by HMBC correlations) with C-2, C-4, C-4a and C-9. The latter correlation is particularly important for the structural assignment as it proves the connection of the quinolone part and the aliphatic bicyclic part of the molecule. Carbon atom C-9 is a quaternary carbon with an unusually high *δ*_C_ value of 91.3 ppm. This suggests a connection to two electronegative atoms, nitrogen atom N-10 and an OH group. The aliphatic bicyclic moiety is related to another *Aristotelia* alkaloid, hobartine (**2**) (Figure 1), where spatial NOE correlations were observed for H-13 (H-12^ax^, H-12^eq^, H-14^ax^, H-19, H-20) and H-15 (H-17, H-14^ax^, H-14^eq^), which provide important information about the configuration of the bicyclic system.

The therapeutic potential of 9-hydroxyaristoquinolone was studied using in silico methods. Compounds with a MW less than 450–500 Da and a TPSA less than 90 Å^2^ have been reported to be associated with higher permeability and are more likely to cross the BBB [34]. 9-Hydroxyaristoquinolone has a MW of 324.424 Da and a TPSA of 65.12 Å^2^. On the other hand, Jose David et al. (2022) corroborated that compounds with log *p* values between 0 and 2 present a higher probability of penetration into the BBB [34,35]. 9-Hydroxyaristoquinolone has a log *p* value (2.75) better than quercetin and galanthin (−7.02 ± 0.08 and −5.35 ± 0.02, respectively). Thus, the BBB permeability of the latter is limited compared to 9-hydroxyaristoquinolone [36]. Furthermore, the absence of significant inhibitory activity on the major CYP450 enzymes, except CYP2D6, minimizes the risk of drug–drug interactions, which supports its initial safety profile according to established preclinical standards [37].

The absence of genotoxicity (AMES assay) and hepatotoxicity reinforces their feasibility, given that many neuroactive agents have failed in late-stage development due to adverse effects of this nature [38]. With zero violations of Lipinski’s rule, 9-hydroxyaristoquinolone exhibits optimal characteristics for oral bioavailability, fundamental in the design of effective neuroactive drugs [39].

The results of morphological analyses in HMC-3 microglial cells demonstrate that 9-hydroxyaristoquinolone effectively inhibits the LPS-induced increase in cell density from concentrations of 1 µM, indicating its ability to prevent microglial activation. This neuroprotective effect may be attributed to the modulation of proinflammatory pathways, which are critical in LPS-mediated processes. Previous studies with similar alkaloids, such as berberine (1 µM) and tetrandrine (30 mg/kg), also evidence anti-inflammatory and neuroprotective effects, inhibiting LPS-induced microglial activation and reducing inflammatory markers, such as IL-1β and TNF-α [40,41,42,43]. These findings demonstrate the relevance of alkaloids as modulators of microglial inflammation in the context of neurodegenerative diseases such as Alzheimer’s and Parkinson’s, in which microglial overstimulation contributes to pathological progression [44,45].

NF-κB plays a key role in the transcriptional regulation of proinflammatory gene expression [46,47,48,49]. Herein, NF-κB-SEAP levels in THP-1XBlue monocytes were used as an effective and reliable methodology to assess the modulatory activity of compounds on the NF-κB pathway. THP1-Blue™ cells were derived from the human monocyte cell line THP-1 by stable integration of an NF-κB-inducible SEAP (secreted embryonic alkaline phosphatase) reporter construct. This SEAP-based reporter system allows quantitative measurement of NF-κB activation in a specific and sensitive manner, facilitating the identification of potential inhibitors [50]. In this context, 9-hydroxyaristoquinolone showed a concentration-dependent inhibitory effect, significantly decreasing SEAP activity at concentrations ≥ 50 µM, reaching a 51.3% reduction at 200 µM. This underscores its ability to attenuate LPS-induced NF-κB activation, a key mediator in inflammation and related neurodegenerative pathologies.

Western blot analysis demonstrated that 9-hydroxyaristoquinolone reduced LPS-induced IκB-α phosphorylation, verifying its ability to inhibit NF-κB inflammatory pathway activation. Pretreatment with 9-hydroxyaristoquinolone at 50 µM, which decreased the P-IκBα/IκBα ratio by 90%, suggests an anti-inflammatory effect that could mitigate neuroinflammatory processes associated with pathologies such as Alzheimer’s and Parkinson’s disease.

## 4. Materials and Methods

### 4.1. General Information

Column chromatography was performed using Merck silica gel 60 and Sephadex LH-20 (25–100 μm; Aldrich, Santiago, Chile). The progress of purification was followed by using analytical thin-layer chromatography (TLC) from Merck Silica Gel 60F254 sheets (Darmstadt, Germany), together with Low-Field NMR (LF-NMR, Bruker 80 Benchtop, Rheinstetten, Germany). TLC was eluted with a mixture of solvents as n-hexane (hex), ethyl acetate (EtOAc) and isopropanol (IsoProp), evaluated by UV light (254 nm) and then stained with Dragendorff and/or with KMnO_4_. Solvents and fractions were concentrated in a rotavap Büchi R100 at 45 °C. Solvents used in this study were distilled prior to use and dried over appropriate drying agents.

### 4.2. Purification of 9-Hydroxyaristoquinolone from Leaves of Aristotelia chilensis

In total, 20 kg of *Aristotelia chilensis* (maqui) leaves were collected in Concepción, at coordinates 36°49′50.72″ S and 73°01′51.07″ W, elevation 73 masl, in January 2019. The plant material was pulverized and macerated for 3 days in water acidified with HCl at pH 3 at room temperature. The aqueous acidic layer was extracted with EtOAc and the organic solvent was evaporated under reduced pressure at 45 °C and 200 mbar to obtain a gummy residue (Total Acid Extract 110 g). The acidic water was then alkalinized to pH 11 with NaHCO_3_-NaOH, and subsequently extracted with EtOAc, 4 L, 3 times. After evaporation of the solvent, 50 g of a rubbery red extract rich in alkaloids was obtained. The crude alkaloid extract was chromatographed using a silica gel column (200–300 mesh) and increasing the solvent polarity from 100% hex to 100% EtOAc to 100% isopropanol, giving fractions F1 to F7. Fraction F6 was purified by CC (60 mesh) eluted in EtOAc/IsoProp (3% *v*/*v*) yielding four subfractions (F-6A to F-6D), which were purified by HPLC using a diol column (10 mm/250 mm). F-6A gave 9-Hydroxyaristoquinolone (32 mg, oily compound, 0.00016% yield).

### 4.3. Chemical Characterization of 9-Hydroxyaristoquinolone

The new alkaloid 9-hydroxyaristoquinolone was fully evaluated by 1D and 2D Nuclear Magnetic Resonance (NMR) spectroscopy. The ^1^H and ^13^C NMR spectra were recorded in CDCl_3_ solution in 5 mm tubes at RT on a Bruker Avance Neo 500 MHz spectrometer (Bruker Biospin GmbH, Rheinstetten, Germany) at 500 (^1^H) and 125 (^13^C) MHz, with the solvent deuterium signal as the shut-off and residual CHCl_3_ (*δ* = 7.26 ppm for ^1^H) or CDCl_3_ (*δ* = 77.16 ppm for ^13^C) for internal calibration. All spectra (^1^H, ^13^C, gs-H,H-COSY, edited HSQC, gs-HMBC and NOESY) were acquired and processed with standard Bruker software TopSpin 4.5.0. High-resolution mass spectra (HRMS) were obtained by EI-TOF (70 eV) using a Waters Micromass instrument. Optical rotations were recorded on a Dichrom model P-2000 polarimeter. IR spectra were recorded as ATR-FTIR spectra using a Perkin-Elmer UART TWO FT-IR-spectrometer (Rodgau, Germany).

### 4.4. Pharmacokinetic and Toxicity Profiles of 9-Hydroxyaristoquinolone

The pharmacokinetics and the toxicity profiles of 9-hydroxyaristoquinolone were predicted using the publicly available SwissADME online server and the ProTox-III platform (https://tox.charite.de/protox3/) accessed on 9 November 2024. The predicted toxicological parameters include molecular weight (MW), number of H-bond acceptor (HBA), H-bond donors (HBD) and rotatable bonds, topological polar surface area (TPSA), partition coefficient (Consensus Log P), solubility class (ESOL), gastrointestinal (GI) absorption, substrate/inhibitor of permeability glycoprotein (P-gp), permeability through the blood–brain barrier (BBB), skin permeability (Log Kp) and susceptibility to metabolic cytochrome P450 enzymes inhibition, all benchmarked with the Lipinski’s rule of five (RO5) accordingly.

### 4.5. Cell Culture

Human microglial cells (HMC-3) were purchased from ATCC (American Type Culture Collection, VA, USA). HMC-3 cells were cultured in Dulbecco’s modified Eagle medium (DMEM) supplemented with 10% fetal bovine serum (FBS) and 0.5% penicillin/streptomycin (all from Cytiva, HYClone Laboratories, UT, USA) in a humidified environment at 37 °C and 5% CO_2_.

THP1-Dual cells (Invivogen, San Diego, CA, USA) were maintained in RPMI-1640 medium with 10% FBS, 25 mM HEPES, 100 μg/mL Normocin, 10 μg/mL Blasticidin, 100 μg/mL Zeocin and 100 μg/mL penicillin/streptomycin solution. THP1 cells were cultured under specific conditions in a humidified atmosphere incubator of 95% air, 5% CO_2_ at 37 °C.

### 4.6. Kinetic Assays of Morphological Changes by Live Cell Analysis -Incucyte^®^

For evaluation of morphological changes, HMC-3 cells were seeded in 96-well plates (5000 cells/well) and incubated at 37 °C and 5% CO_2_ for 24 h in RPMI 1640 medium supplemented with 10% SFB. A stock solution of 9-hydroxyaristoquinolone (50,000 µM in DMSO) was prepared, and dilutions were made in RPMI for treatments at concentrations of 50, 25, 6.25 and 1 µM. The cells were pre-incubated for 2 h with the alkaloid and then Lipopolysaccharide (LPS 100 ng/mL) was added. The plates were placed in the IncuCyte S3 real-time monitoring system, which was configured to acquire 2 images per well with the 10× objective, in phase contrast and FITC channel, every 1 or 2 h for 24 h. Kinetic analyses of confluence (%) and object count per well were performed automatically every 1 or 2 h with the “cell-by-cell” processing and segmentation module (Sartorius # 9600-0031), continuously over time.

### 4.7. Detection of NF-κB-SEAP Levels by THP-1-Dual Cells

The THP1-Dual cells (invivogen) cell line, derived from human monocytes, in which the enzyme alkaline phosphatase is expressed under the control of the NF-kB transcriptional factor promoter, was used for this study. For this purpose, ~100,000 THP-1 cells per well were seeded in a 96-well plate, and then the cells were pre-incubated with 9-hydroxyaristoquinolone at concentrations of 12.5, 25, 50, 100 and 200 µM by 1 h. Then, the plates were incubated with LPS (100 ng/mL × 18 h) at 37 °C, 5% CO_2_. After this time, 180 µL of QUANTI-Blue™ Solution per well was added to another 96-well plate, along with 20 µL of the previously treated cell suspension. SEAP levels were determined after 30 min using a spectrophotometer at 630 nm. The expression of NF-κB activity results is performed with respect to the positive control, cells treated with LPS, using the following formula:

% NF-κB activity = (Abs 630 nm sample/Abs 630 nm LPS control) × 100.

### 4.8. Western Blot Assay on the Modulation of Nuclear Factor NF-κB

HMC-3 cells (1 × 10^6^ cells/well) were seeded and treated for 3 h with 50 µM of 9-hydroxyaristoquinolone with and without LPS (2 h with 100 ng/mL). After a total of 5 h of incubation, the cells were washed with precooled PBS, twice. Cells were lysed with RIPA Lysis Buffer System^®^ (#sc-24948, Santa Cruz, Dallas, TX, USA) containing protease and phosphatase inhibitors. Cells were scraped and transferred to 1.5 mL tubes and centrifuged at 12,000× *g* for 20 min. The supernatant was collected, and protein concentration was measured using a Pierce™ BCA Protein Assay Kit (#23227, Thermo Scientific™, Waltham, Massachusetts, USA). Subsequently, 30 µg of proteins was separated by electrophoresis and transferred to a PVDF (polyvinylidene fluoride) membrane. The membrane was incubated overnight with the primary antibody in Tris-buffered saline and Tween20^®^ at 4 °C. Antibodies against IκBα (1:1000, no. 4812, Cell signaling, Danvers, MA, USA), Phospho-IκBα (1:1000, no. 9246, Cell signaling) and a-tubulin (1:1000, no. sc-5286, Santa Cruz) were used. Immunoreactive bands were visualized using a horseradish peroxidase-conjugated secondary antibody (Anti-Mouse IgG, no. 715-035-150; Anti-Rabbit IgG 711-035-152, 1:10,000, Jackson Immunoresearch, West Grove, PA, USA) and chemiluminescence detection with SuperSignal™ West Pico PLUS chemiluminescent substrate (no. 34579, Thermo Scientific™). Images were obtained with the G:Box chemi XRQ gel doc system (Syngene, Cambridge, UK) and band densitometry analysis was performed with Image J software V.1.49 (NIH, Bethesda, MD, USA).

## 5. Conclusions

9-Hydroxyaristoquinolone is a bioactive compound found in the medicinal tree *Aristotelia chilensis*. In this study, it was shown to inhibit the NF-κB signaling pathway by preventing IκBα phosphorylation in HMC-3 cells, an in vitro model of activated microglia. These findings suggest that 9-hydroxyaristoquinolone could be a promising candidate for modulating neuroinflammation. However, further research is needed to elucidate its precise mechanisms of action and assess its potential for clinical applications.

## Figures and Tables

**Table 1:**
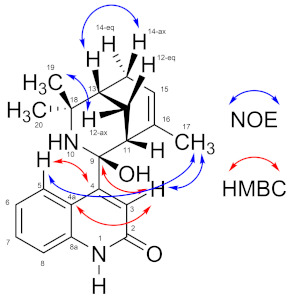
NMR spectroscopic data (^1^H 500 MHz, ^13^C 125 MHz, CDCl_3_) for 9-Hydroxyaristoquinolone.

Position	*δ*_H_ m (*J* in Hz)	*δ*_C_, Type	HMBC	NOE
1	n.d.	--		
2	--	174.0, C		
3	5.90 s	117.9, CH	C-2, C-4, C-4a, C-9	H-17
4	--	162.0, C		
4a	--	120.9, C		
5	7.44 d (7.6)	123.9, CH	C-4, C-7, C-8a	H-17
6	6.91 ddd (7.6, 7.6, 1.0)	121.3, CH	C-4a, C-8	
7	7.24 ddd (8.0, 7.6, 1.2)	131.5, CH	C-5, C-8a	
8	6.85 d (8.0)	112.9, CH	C-4a, C-6	
8a	--	154.4, C		
9	--	91.3, C		
10	n.d.	--		
11	2.12 dd (2.8, 2.8)	45.7, CH		
12	ax: 2.45 ddd (13.7, 3.0, 3.0)eq: 1.87 ddd (13.7, 2.9, 2.9)	25.0, CH_2_	C-16C-9, C-18	
13	1.67 m	37.8, CH	C-15	H-12^ax^, H-12^eq^, H-14^ax^, H-19, H-20
14	eq: 2.64 d (19.3)ax: 2.15 dm (19.3)	27.5, CH_2_	C-15	H-20H-13
15	5.49 m	125.2, CH		H-17, H-14^ax^, H-14^eq^
16	--	131.6, C		
17	1.51 m	24.4, CH_3_	C-16, C-15, C-11	H-3, H-5, H-11, H-15
18	--	58.9, C		
19	1.45 s	29.6, CH_3_	C-18, C-13, C-20	
20	1.89 s	26.9, CH_3_	C-18, C-13, C-19	

**Table 2:** Pharmacokinetic properties, pharmacodynamic characteristics and predicted toxicity for the compound 9-hydroxyaristoquinolone, evaluated using in silico tools. Parameters include molecular weight (MW), number of rotatable bonds, #H-bond acceptors/donors, polar surface area (TPSA), solubility (ESOL Class), blood–brain barrier permeability (BBB permeant) and toxicity predictions such as skin sensitization and cardiotoxicity (hERG I/II inhibitors).

Pharmacokinetics/Druglikeness			
Entry	9-Hydroxyaristoquinolone	Toxicity	9-Hydroxyaristoquinolone
MW	324.424	AMES toxicity	No
#Rotatable bonds	1	Hepatotoxicity	No
#H-bond acceptors	3	hERG I/II inhibitors	No/Yes
#H-bond donors	3	Skin sensitization	No
TPSA	65.12		
Consensus Log Po/w	2.75		
ESOL Class	Soluble		
GI absorption	High		
BBB permeant	Yes		
Pgp substrate	Yes		
CYP1A2 inhibitor	No		
CYP2C19 inhibitor	No		
CYP2C9 inhibitor	No		
CYP2D6 inhibitor	Yes		
CYP3A4 inhibitor	No		
log Kp (cm/s)	−7.29		
Lipinski #violations	0		
Synthetic Accessibility	4.67		

## Data Availability

The original data presented in the study are openly available at https://doi.org/10.5281/zenodo.14652284.

## Supplementary Materials

The following supporting information can be downloaded at: https://www.mdpi.com/article/10.3390/ijms26062419/s1.

## Abbreviations

AD, Alzheimer’s disease; Aβ, amyloid-β; TLR, Toll-like receptors; NF-Κb, nuclear factor kappa-light-chain-enhancer of activated B cells; TNF-α, tumor necrosis factor; IKK, IκB kinase; IκB, inhibitory κB; IκBα-P, phosphorylated IκBα; IL, interleukin; COX2, cyclooxygenase-2; CCL1, chemokine ligand 1; CXCL1, CXC motif ligand 1; TNFR2, tumor necrosis factor receptor 2; MAP, Mitogen-Activated Protein; NLRP3, NOD-, LRR-, and Pyrin domain-containing protein 3; TLC, thin-layer chromatography; hex, n-hexane; EtOAc, ethyl acetate; NMR, Nuclear Magnetic Resonance; MW, molecular weight; HBA, number of H-bond acceptor, HBD, H-bond donors; TPSA, topological polar surface area; Consensus Log P, partition coefficient; ESOL, solubility class; GI, gastrointestinal; P-gp, substrate/inhibitor of permeability glycoprotein; BBB, permeability through the blood-brain barrier; Log Kp, skin permeability; RO5, Lipinskis rule of five; HMC-3, Human microglial cells; DMEM, Dulbecco’s modified Eagle medium; FBS, fetal bovine serum; THP-1, Human monocytic; LPS, Lipopolysaccharide; SEAP, secreted embryonic alkaline phosphatase.

## References

[1] Cao X., Hou Y., Zhang X., Xu C., Jia P., Sun X., Sun L., Gao Y., Yang H., Cui Z. (2020). A comparative, correlate analysis and projection of global and regional life expectancy, healthy life expectancy, and their GAP: 1995–2025. J. Glob. Health.

[2] Naciones Unidas Desafios Globales adlphwuoeg-ipcd. https://www.un.org/development/desa/pd/es/content/World-Population-Prospects-2022.

[3] Hou Y., Dan X., Babbar M., Wei Y., Hasselbalch S.G., Croteau D.L., Bohr V.A. (2019). Ageing as a risk factor for neurodegenerative disease. Nat. Rev. Neurol..

[4] Zhang X.X., Tian Y., Wang Z.T., Ma Y.H., Tan L., Yu J.T. (2021). The Epidemiology of Alzheimer’s Disease Modifiable Risk Factors and Prevention. J. Prev. Alzheimer’s Dis..

[5] Miranda A., Montiel E., Ulrich H., Paz C. (2021). Selective Secretase Targeting for Alzheimer’s Disease Therapy. J. Alzheimer’s Dis..

[6] Guzman-Martinez L., Maccioni R.B., Andrade V., Navarrete L.P., Pastor M.G., Ramos-Escobar N. (2019). Neuroinflammation as a Common Feature of Neurodegenerative Disorders. Front. Pharmacol..

[7] Hampel H., Caraci F., Cuello A.C., Caruso G., Nisticò R., Corbo M., Baldacci F., Toschi N., Garaci F., Chiesa P.A. (2020). A Path Toward Precision Medicine for Neuroinflammatory Mechanisms in Alzheimer’s Disease. Front. Immunol..

[8] Doens D., Fernández P.L. (2014). Microglia receptors and their implications in the response to amyloid β for Alzheimer’s disease pathogenesis. J. Neuroinflamm..

[9] García-Revilla J., Alonso-Bellido I.M., Burguillos M.A., Herrera A.J., Espinosa-Oliva A.M., Ruiz R., Cruz-Hernández L., García-Domínguez I., Roca-Ceballos M.A., Santiago M. (2019). Reformulating Pro-Oxidant Microglia in Neurodegeneration. J. Clin. Med..

[10] Pampuscenko K., Morkuniene R., Krasauskas L., Smirnovas V., Brown G.C., Borutaite V. (2023). Extracellular tau stimulates phagocytosis of living neurons by activated microglia via Toll-like 4 receptor–NLRP3 inflammasome–caspase-1 signalling axis. Sci. Rep..

[11] Hopp S.C., Lin Y., Oakley D., Roe A.D., DeVos S.L., Hanlon D., Hyman B.T. (2018). The role of microglia in processing and spreading of bioactive tau seeds in Alzheimer’s disease. J. Neuroinflamm..

[12] Patel J.R., Brewer G.J. (2008). Age-related changes to tumor necrosis factor receptors affect neuron survival in the presence of beta-amyloid. J. Neurosci. Res..

[13] Streit W.J., Braak H., Del Tredici K., Leyh J., Lier J., Khoshbouei H., Eisenlöffel C., Müller W., Bechmann I. (2018). Microglial activation occurs late during preclinical Alzheimer’s disease. Glia.

[14] Disabato D.J., Quan N., Godbout J.P. (2016). Neuroinflammation: The devil is in the details. J. Neurochem..

[15] Lyu J., Jiang X., Leak R.K., Shi Y., Hu X., Chen J. (2021). Microglial Responses to Brain Injury and Disease: Functional Diversity and New Opportunities. Transl. Stroke Res..

[16] Rodríguez-Gómez J.A., Kavanagh E., Engskog-Vlachos P., Engskog M.K., Herrera A.J., Espinosa-Oliva A.M., Joseph B., Hajji N., Venero J.L., Burguillos M.A. (2020). Microglia: Agents of the CNS Pro-Inflammatory Response. Cells.

[17] Anilkumar S., Wright-Jin E. (2024). NF-κB as an Inducible Regulator of Inflammation in the Central Nervous System. Cells.

[18] Sivamaruthi B.S., Raghani N., Chorawala M., Bhattacharya S., Prajapati B.G., Elossaily G.M., Chaiyasut C. (2023). NF-κB Pathway and Its Inhibitors: A Promising Frontier in the Management of Alzheimer’s Disease. Biomedicines.

[19] Muñoz O., Christen P., Cretton S., Backhouse N., Torres V., Correa O., Costa E., Miranda H., Delporte C. (2011). Chemical study and anti-inflammatory, analgesic and antioxidant activities of the leaves of *Aristotelia chilensis* (Mol.) Stuntz, Elaeocarpaceae. J. Pharm. Pharmacol..

[20] Rubilar M., Jara C., Poo Y., Acevedo F., Gutierrez C., Sineiro J., Shene C. (2011). Extracts of Maqui (*Aristotelia chilensis*) and Murta (*Ugni molinae* Turcz.): Sources of antioxidant compounds and α-Glucosidase/α-Amylase inhibitors. J. Agric. Food Chem..

[21] Velázquez L., Quiñones J., Inostroza K., Sepúlveda G., Díaz R., Scheuermann E., Domínguez R., Lorenzo J.M., Velásquez C., Sepúlveda N. (2022). Maqui (*Aristotelia chilensis* (Mol.) Stuntz): A Natural Antioxidant to Improve Quality of Meat Patties. Antioxidants.

[22] Cespedes C., Jakupovic J., Silva M., Watson W. (1990). Indole alkaloids from Aristotelia chilensis. Phytochemistry.

[23] Cifuentes F., Palacios J., Paredes A., Nwokocha C.R., Paz C. (2018). 8-Oxo-9-Dihydromakomakine Isolated from *Aristotelia chilensis* Induces Vasodilation in Rat Aorta: Role of the Extracellular Calcium Influx. Molecules.

[24] Pérez R., Figueredo C., Burgos V., Cabrera-Pardo J.R., Schmidt B., Heydenreich M., Koch A., Deuis J.R., Deuis J.R., Paz C. (2023). Natural Compounds Purified from the Leaves of *Aristotelia chilensis*: Makomakinol, a New Alkaloid and the Effect of Aristoteline and Hobartine on Na_V_ Channels. Int. J. Mol. Sci..

[25] Romero F., Palacios J., Jofré I., Paz C., Nwokocha C.R., Paredes A., Cifuentes F. (2019). Aristoteline, an Indole-Alkaloid, Induces Relaxation by Activating Potassium Channels and Blocking Calcium Channels in Isolated Rat Aorta. Molecules.

[26] Argade M.D., Riley A.P. (2022). Syntheses of Aristotelia Alkaloids: Reflections in the Chiral Pool. Synlett Acc. Rapid Commun. Synth. Org. Chem..

[27] Argade M.D., Straub C.J., Rusali L.E., Santarsiero B.D., Riley A.P. (2021). Synthesis of Aristoquinoline Enantiomers and Their Evaluation at the α3β4 Nicotinic Acetylcholine Receptor. Org. Lett..

[28] Arias H.R., Ortells M.O., Feuerbach D., Burgos V., Paz C. (2019). Alkaloids Purified from Aristotelia chilensis Inhibit the Human α3β4 Nicotinic Acetylcholine Receptor with Higher Potencies Compared with the Human α4β2 and α7 Subtypes. J. Nat. Prod..

[29] Dobler M., Borschberg H.-J., Azerad R. (1995). Microbial hydroxylation of some synthetic Aristotelia alkaloids. Tetrahedron Asymmetry.

[30] Schön C., Wacker R., Micka A., Steudle J., Lang S., Bonnländer B. (2018). Bioavailability Study of Maqui Berry Extract in Healthy Subjects. Nutrients.

[31] Zúñiga G.E., Tapia A., Arenas A., Contreras R.A., Zúñiga-Libano G. (2017). Phytochemistry and biological properties of Aristotelia chilensis a Chilean blackberry: A review. Phytochem. Rev..

[32] Bribiesca-Cruz I., Moreno D.A., García-Viguera C., Gallardo J.M., Segura-Uribe J.J., Pinto-Almazán R., Guerra-Araiza C. (2021). Maqui berry (*Aristotelia chilensis*) extract improves memory and decreases oxidative stress in male rat brain exposed to ozone. Nutr. Neurosci..

[33] Cespedes C.L., Balbontin C., Avila J.G., Dominguez M., Alarcon J., Paz C., Burgos V., Ortiz L., Peñaloza-Castro I., Seigler D.S. (2017). Inhibition on cholinesterase and tyrosinase by alkaloids and phenolics from *Aristotelia chilensis* leaves. Food Chem. Toxicol. Int. J. Publ. Br. Ind. Biol. Res. Assoc..

[34] Hitchcock S.A. (2008). Blood–brain barrier permeability considerations for CNS-targeted compound library design. Curr. Opin. Chem. Biol..

[35] Waterhouse R. (2003). Determination of lipophilicity and its use as a predictor of blood–brain barrier penetration of molecular imaging agents. Mol. Imaging Biol..

[36] Könczöl Á., Müller J., Földes E., Béni Z., Végh K., Kéry Á., Balogh G.T. (2013). Applicability of a blood–brain barrier specific artificial membrane permeability assay at the early stage of natural product-based CNS drug discovery. J. Nat. Prod..

[37] Ogu C.C., Maxa J.L. (2000). Drug interactions due to cytochrome P450. Bayl. Univ. Med. Cent. Proc..

[38] Vukotić N.T., Đorđević J., Pejić S., Đorđević N., Pajović S.B. (2021). Antidepressants- and antipsychotics-induced hepatotoxicity. Arch. Toxicol..

[39] Ivanović V., Rančić M., Arsić B., Pavlović A. (2020). Lipinski’s rule of five, famous extensions and famous exceptions. Pop. Sci. Artic..

[40] Kim M., Shin M.S., Lee J.M., Cho H.S., Kim C.J., Kim Y.J., Choi H.R., Jeon J.W. (2014). Inhibitory Effects of Isoquinoline Alkaloid Berberine on Ischemia-Induced Apoptosis via Activation of Phosphoinositide 3-Kinase/Protein Kinase B Signaling Pathway. Int. Neurourol. J..

[41] Lu D., Tang C., Chen Y., Wei I. (2010). Berberine suppresses neuroinflammatory responses through AMP-activated protein kinase activation in BV-2 microglia. J. Cell. Biochem..

[42] Wang H., Liu C., Mei X., Cao Y., Guo Z., Yuan Y., Zhao Z., Song C., Guo Y., Shen Z. (2017). Berberine attenuated pro-inflammatory factors and protect against neuronal damage via triggering oligodendrocyte autophagy in spinal cord injury. Oncotarget.

[43] Wang J., Guo M., Ma R., Wu M., Zhang Y. (2020). Tetrandrine alleviates cerebral ischemia/reperfusion injury by suppressing NLRP3 inflammasome activation via Sirt-1. PeerJ.

[44] Azam S., Haque E., Kim I.-S., Choi D.-K. (2021). Microglial Turnover in Ageing-Related Neurodegeneration: Therapeutic Avenue to Intervene in Disease Progression. Cells.

[45] Fan Z., Aman Y., Ahmed I., Chetelat G., Landeau B., Chaudhuri K.R., Brooks D.J., Edison P. (2015). Influence of microglial activation on neuronal function in Alzheimer’s and Parkinson’s disease dementia. Alzheimer’s Dement. J. Alzheimer’s Assoc..

[46] Aggarwal B.B., Takada Y., Shishodia S., Gutierrez A.M., Oommen O.V., Ichikawa H., Baba Y., Kumar A. (2004). Nuclear transcription factor NF-kappa B: Role in biology and medicine. Indian J. Exp. Biol..

[47] Lappas M., Permezel M., Georgiou H.M., Rice G.E. (2002). Nuclear factor kappa B regulation of proinflammatory cytokines in human gestational tissues in vitro. Biol. Reprod..

[48] Oeckinghaus A., Ghosh S. (2009). The NF-κB family of transcription factors and its regulation. Cold Spring Harb. Perspect. Biol..

[49] Saegusa M., Hashimura M., Kuwata T. (2010). Pin1 acts as a modulator of cell proliferation through alteration in NF-κB but not β-catenin/TCF4 signalling in a subset of endometrial carcinoma cells. J. Pathol..

[50] Validation Data for THP1-Blue™ NF-κB Cells. https://www.invivogen.com/thp1-blue-nfkb.

