# Supplementary material for: 9-Hydroxyaristoquinolone: A New Indole Alkaloid Isolated from Aristotelia chilensis with Inhibitory Activity of NF-κB in HMC-3 Microglia Cells
Source: Int J Mol Sci. 2025 Mar 7;26(6):2419. doi: 10.3390/ijms26062419 (PMC11942453; doi:10.3390/ijms26062419)

Supporting Information

9-Hydroxyaristoquinolone a new indole alkaloid isolated from *Aristotelia chilensis* with inhibitory activity of NF-kB in HMC-3 microglia cells

Rebeca Pérez [1], Viviana Burgos [2], Jaime R. Cabrera-Pardo [3,4], Leandro Ortiz [5], Antonio Camins [6], Miren Ettcheto [6], Bernd Schmidt [7], Vaderament-A. Nchiozem-Ngnitedem [7] and Cristian Paz [1*]

1. Laboratory of Natural Products & Drug Discovery, Center CEBIM, Department of Basic Sciences, Faculty of Medicine, Universidad de La Frontera, Temuco 4780000, Chile. perezcolladorebeca@gmail.com (R.P.)
2. Escuela de Tecnología Médica, Facultad de Salud, Universidad Santo Tomás, Chile; vburgos7@santotomas.cl (V.B.)
3. Laboratorio de Química Aplicada y Sustentable (LabQAS), Departamento de Química, Universidad del Bío-Bío, Avenida Collao 1202, Concepción 4051381, Chile; jacabrera@ubiobio.cl (J.R.C-P.)
4. College of Dental Medicine, Roseman University of Health Sciences, 10894 S. River Front Parkway, South Jordan, UT 84095, USA; jcabrerapardo@roseman.edu (J.R.C-P.)
5. Instituto de Ciencias Químicas, Facultad de Ciencias, Universidad Austral de Chile, Valdivia 5110566, Chile; leandro.ortiz@uach.cl
6. Departament de Farmacologia, Toxicologia i Química Terapèutica, Facultat de Farmàcia i Ciències de l'Alimentació, Universitat de Barcelona (UB), Av. de Joan XXIII, 27-31, Barcelona 08028, Spain; Institut de Neurociències, Universitat de Barcelona (UB), Passeig de la Vall d'Hebron, 171, Barcelona 08035, Spain; Centro de Investigación Biomédica en Red Enfermedades Neurodegenerativas (CIBERNED), Instituto de Carlos III, Av. Monforte de Lemos, 3-5, Madrid 28029, Spain; Institut d'Investigació Sanitària Pere Virgili (IISPV), Hospital Universitari Sant Joan de Reus, Av. Josep Laporte, 2, Reus 43204, Spain. camins@ub.edu (A.C.); mirenettcheto@ub.edu (M.E.)
7. Institut für Chemie, Universität Potsdam, Karl-Liebknecht-Str. 24-25, Potsdam D-14476, Germany. bernd.schmidt@uni-potsdam.de (B.S.); n.vaderamentalexe@gmail.com (V.A.N-N.)

* Correspondence: cristian.paz@ufrontera.cl; Tel.: +56 45 259 2825.

Academic Editor: Firstname Lastname

Received: date
Revised: date
Accepted: date
Published: date

Citation: To be added by editorial staff during production.

Copyright: © 2025 by the authors. Submitted for possible open access publication under the terms and conditions of the Creative Commons Attribution (CC BY) license (https://creativecommons.org/licenses/by/4.0/).

Contents:

NMR-Data assignment for 9-hydroxyaristoquinolone
Structure of 9-hydroxyaristoquinolone with numbering scheme
Copies of 1D- and 2D-NMR spectra

Figure S1: ^{1}H NMR (500 MHz, $CDCl_3$) of 9-Hydroxyaristoquinolone (**3**)

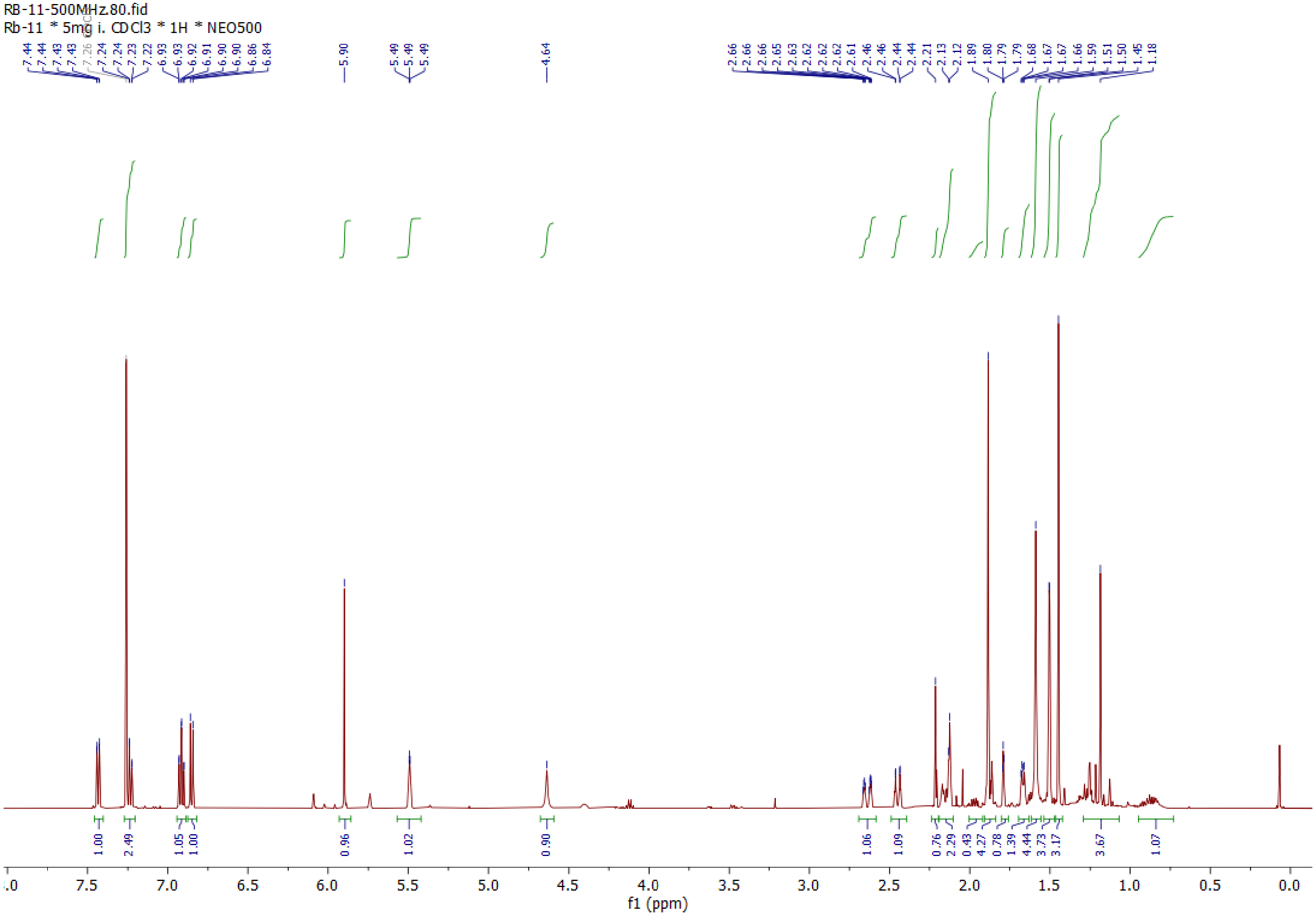

Figure S2: $^{13}C\{^1H\}$ NMR (125 MHz, $CDCl_3$) of 9-Hydroxyaristoquinolone (**3**)

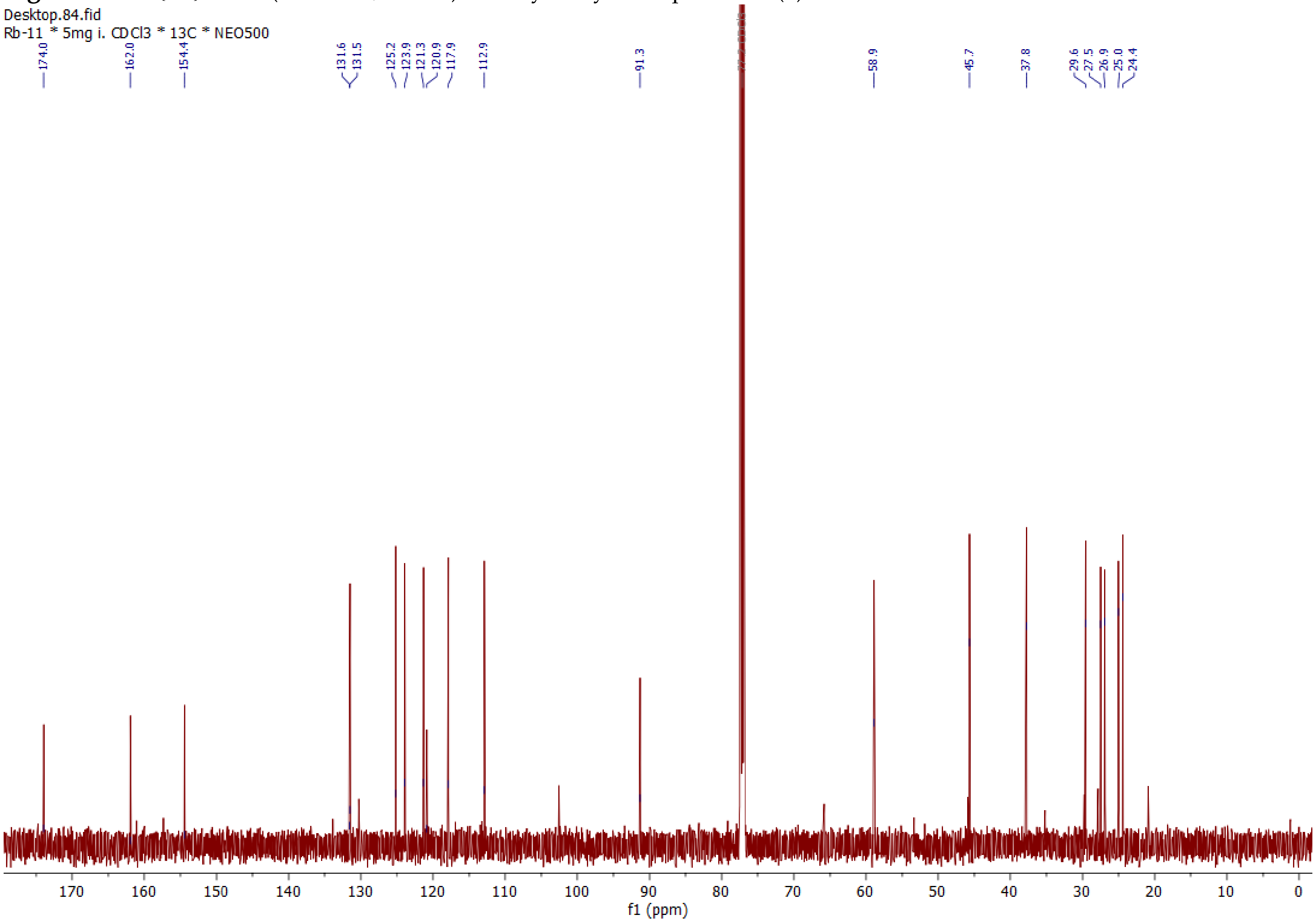

Figure S3: H,H-COSY (500 MHz, $CDCl_3$) of 9-Hydroxyaristoquinolone (**3**)

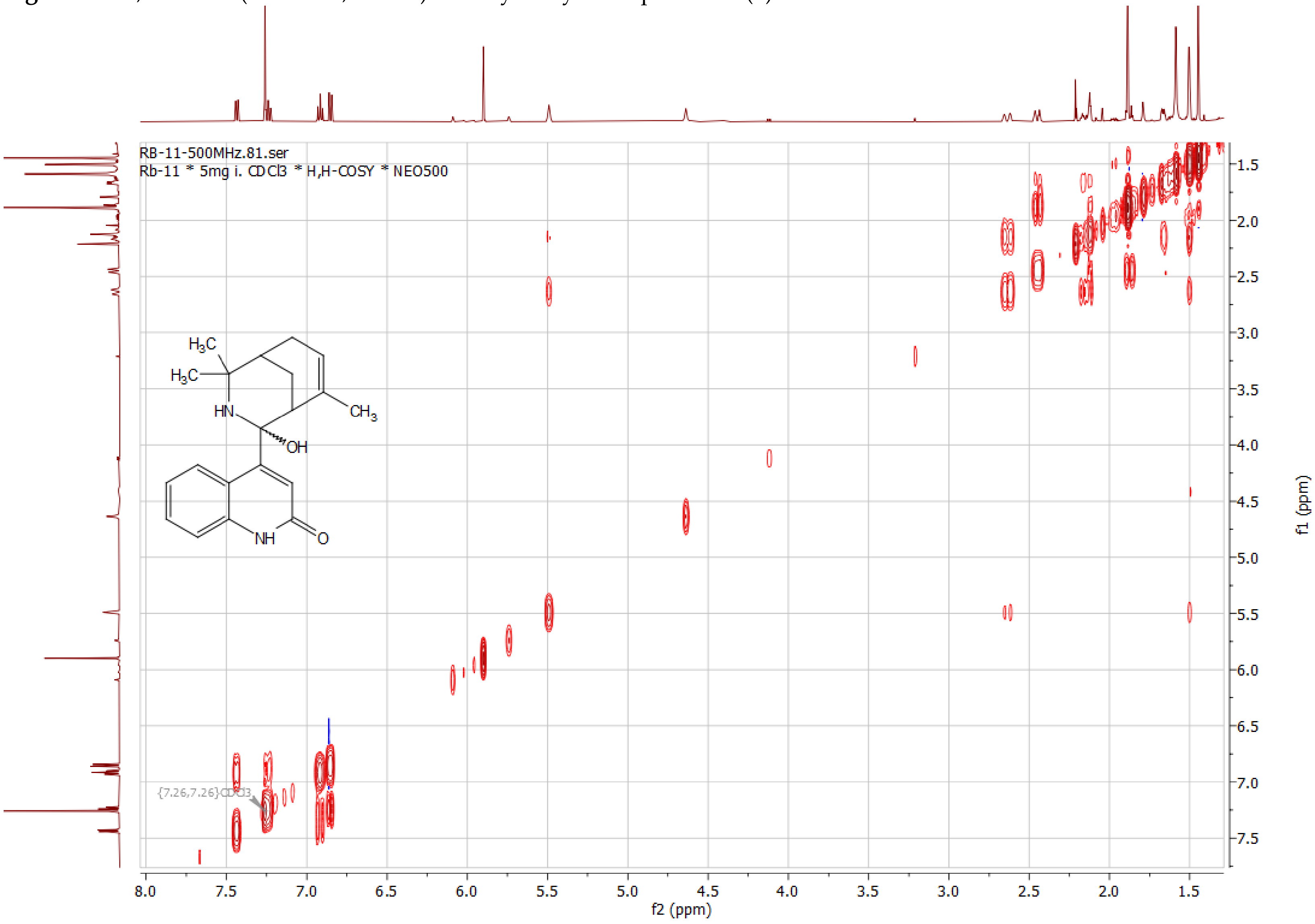

Figure S4: HSQC (500/125 MHz, CDCl3) of 9-Hydroxyaristoquinolone (**3**)

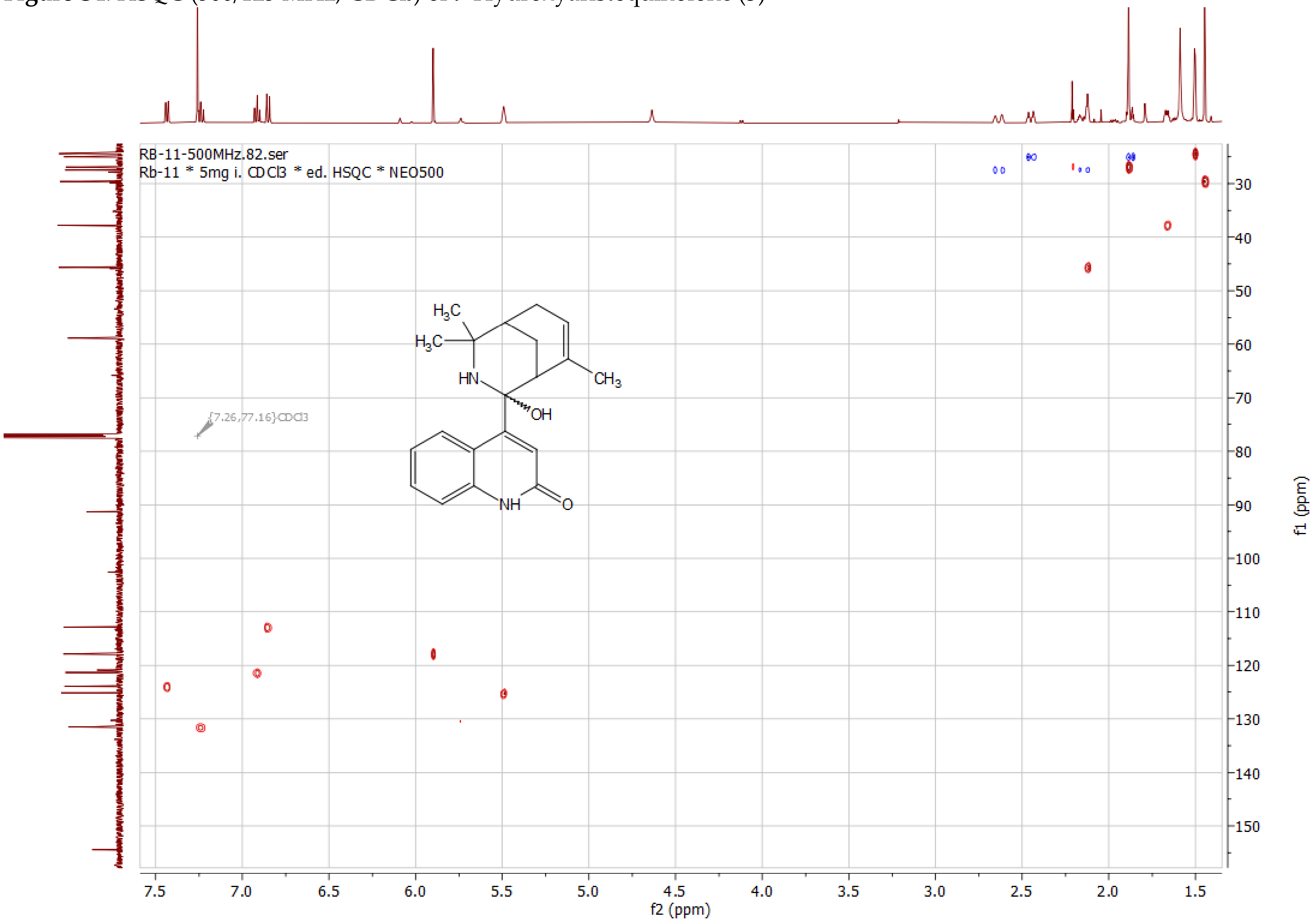

Figure S5: HMBC (500/125 MHz, $CDCl_3$) of 9-Hydroxyaristoquinolone (**3**)

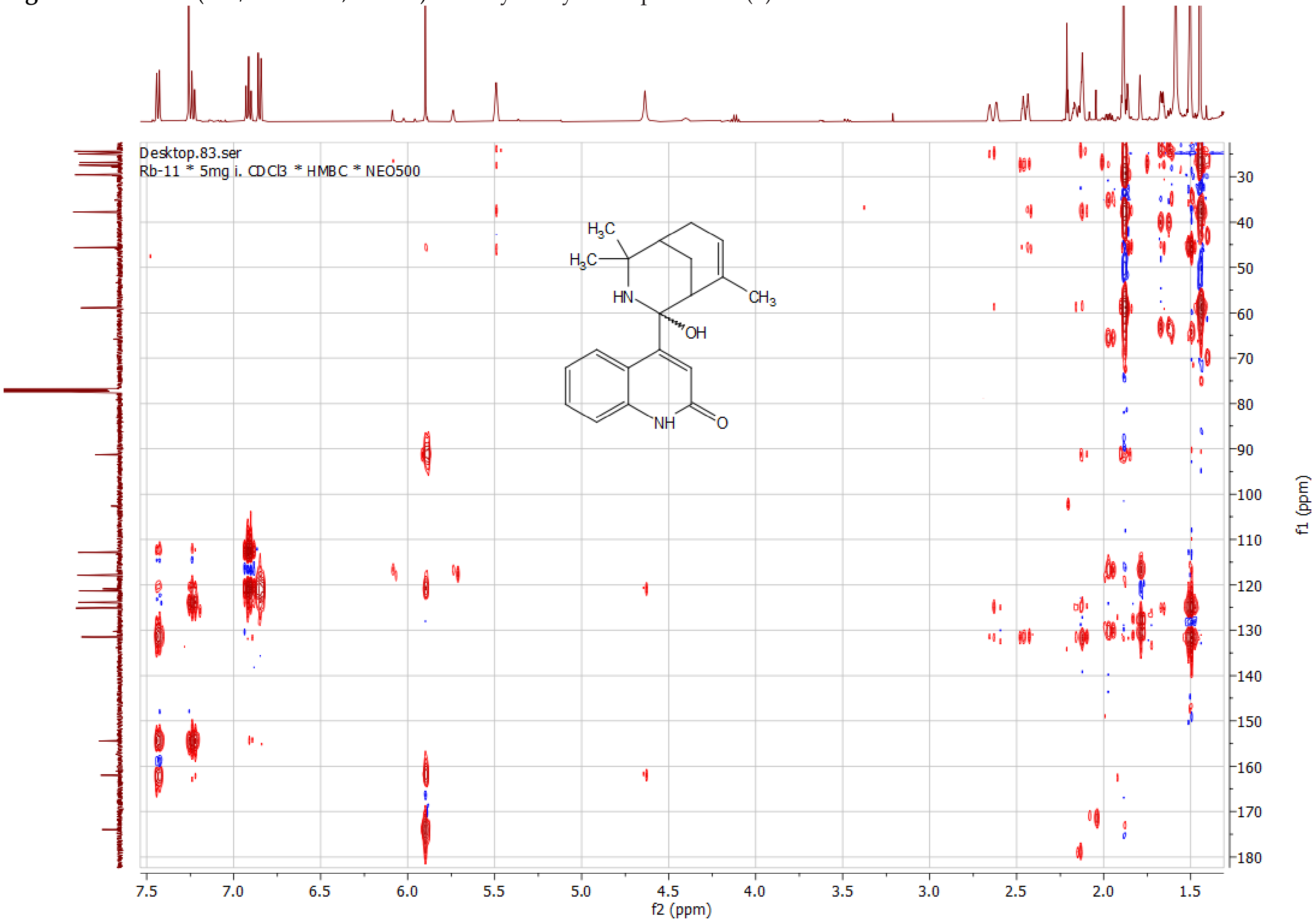

Figure S6: NOESY (500 MHz, $CDCl_3$) of 9-Hydroxyaristoquinolone (**3**)

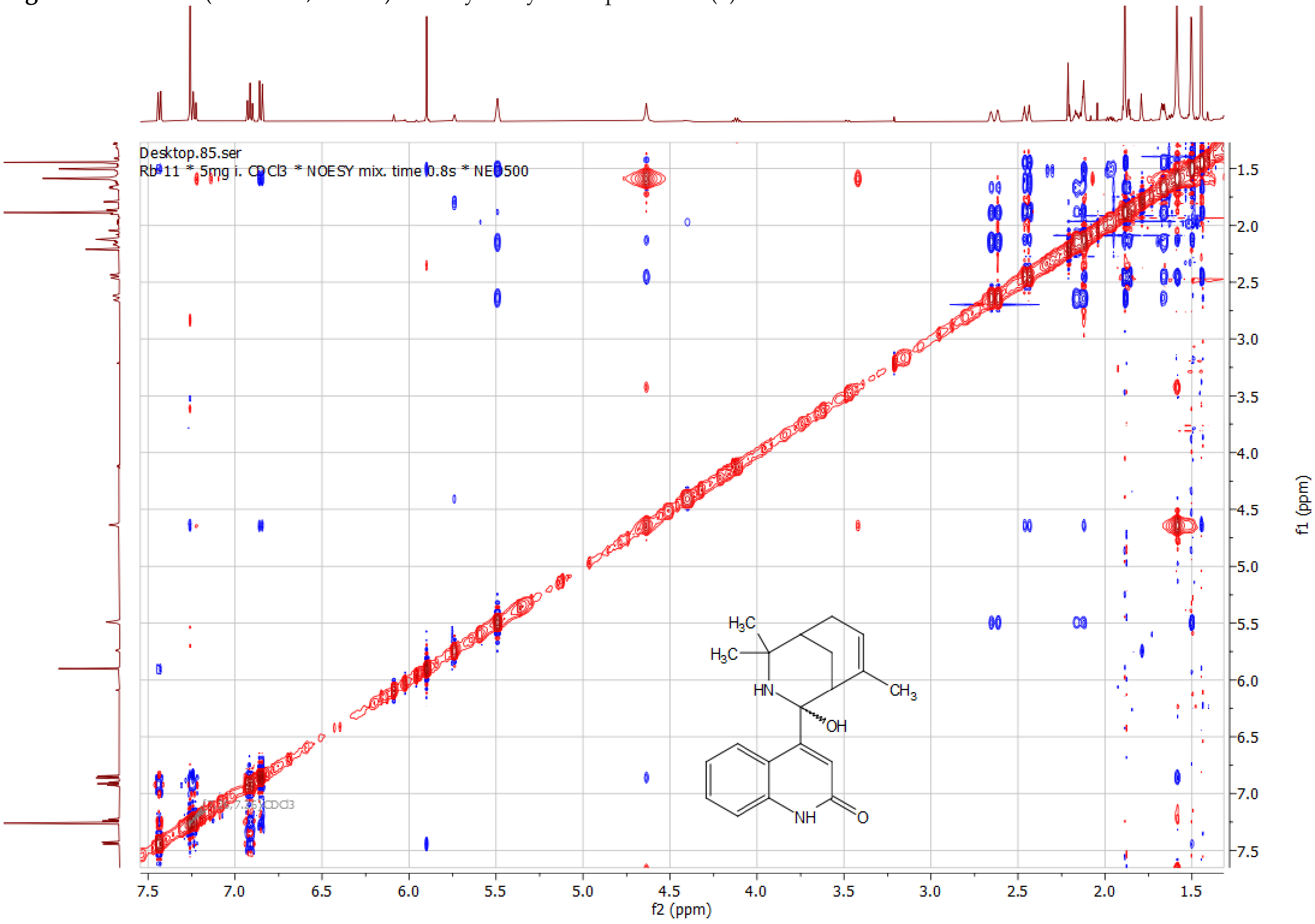

Supplement: Supplementary file 1 [file ijms-26-02419-s001.zip › ijms-3468768-supplementary.pdf]
